# Comparison of CBCT Prescriptions among Different Campuses of East Carolina University School of Dental Medicine

**DOI:** 10.1155/2022/2754174

**Published:** 2022-09-13

**Authors:** Cody C. Phen, Gerard A. Camargo, Wenjian Zhang

**Affiliations:** East Carolina University School of Dental Medicine, 1851 MacGregor Downs Road, Greenville, NC 27834, USA

## Abstract

**Objectives:**

East Carolina University School of Dental Medicine (ECU SoDM) has established a unique education model that delivers the dental curriculum through student rotations at community service learning centers (CSLCs) in underserved areas across North Carolina in their senior year. The objective of the study is to analyze the patient composition and CBCT prescription patterns at the main campus (Ross Hall) and eight CSLCs, to determine if students have comparable training at various sites.

**Methods:**

CBCTs taken at ECU SoDM in 2017–2021 were evaluated. One-way analysis of variance and the Wilcoxon Rank Sum Test were used to determine any differences in demographics, Medicaid coverage, and scan indications at various sites.

**Results:**

A total of 1444 patients were included, with an age range of 4–90 years old; male 685, female 758; 1130 non-Hispanic/Latino, 71 Hispanic/Latino. For races, Caucasian 1106, African-American 156, American Indian/Alaskan Native 32, Asian 18, mixed 13, other 73. For Medicaid, there are 75 with and 1369 without coverage. Ross Hall has the largest amount of patients at 537, followed by Davidson 218, Brunswick 189, and Lillington 169, with Elizabeth City being the least with 45 patients. The top four reasons for taking CBCTs were implants, endodontics, oral pathology, and 3rd molar assessment. There was no significant difference in the indications for CBCTs or Medicaid coverage, among various sites.

**Conclusions:**

The demographics, Medicaid coverage and CBCT prescription patterns were comparable among various sites. There was a site-related difference in the amount of scans taken, warranting the necessity to monitor CSLC rotation selections to ensure a consistent learning experience.

## 1. Introduction

East Carolina University School of Dental Medicine (ECU SoDM), North Carolina's second dental school, was established to address the shortage of dentists in the rural regions across North Carolina. It enrolled its inaugural class in 2011. The campus includes the main teaching facility, Ledyard E. Ross Hall on ECU's Health Sciences Campus in Greenville, and eight community service learning centers (CSLCs) in rural and underserved locations, including Ahoskie, Brunswick, Elizabeth City, Davidson, Lillington, Robeson, Spruce Pine, and Sylva. ECU SoDM has developed a unique model of oral health education via the incorporation of traditional dental curriculum with community-based, service-learning, and outreach, leading the nation in the cultivation of future dentists committed to addressing oral health needs in the underserved, rural, and disadvantaged communities.

According to the curriculum of ECU SoDM, the fourth year is divided into five 9-week clinical rotations, two at Ross Hall and three at CSLCs, therefore, the rotations at CSLCs account for 60% of students' clinical experience in their final year. Through rotations, students provide much-needed oral health care to the rural communities of all 100 counties in the state, while sharpening dental skills as well as gaining experience in business and practice management.

Due to differences in the geographical, cultural, and social characteristics of each community, the oral health care needs of each CSLC could vary greatly, which may contribute to inconsistence of learning and patient care experience for the students.

The objective of the current study is to analyze the patient composition and CBCT prescription patterns at Ross Hall and eight CSLCs, to determine if students have similar 3D imaging-related learning experiences at various campus sites.

## 2. Materials and Methods

CBCTs taken at Ross Hall and eight CSLCs from June 2017 to June 2021 were evaluated. The demographic information (age, gender, ethnicity, and race), Medicaid coverage, and reasons for the scans were collected from Axium, the school's electronic health record system. Basically, all of the patients who had CBCT procedure codes completed during the time interval were screened, and their demographic information, including age, gender, ethnicity, and race were recorded. Their CBCT acquisition forms were consulted for reasons for taking the scans. Their Medicaid coverage information was retrieved from the “Transaction” section of the Axium.

Depending on the distribution of data, a one-way analysis of variance (for normally distributed data) or a Wilcoxon Rank Sum Test (for nonnormal distributed data) was run to determine any significant difference in the demographics, Medicaid coverage, and scan indications among various campus locations. An IRB exemption was obtained from the institution prior to the start of the project (UMCIRB21-001253).

## 3. Results

**Figure 1:**
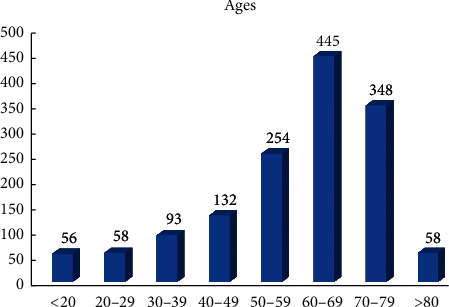
Ages of the patients. The numbers on top of the bars represent the patient counts in each age range category.

**Figure 2:**
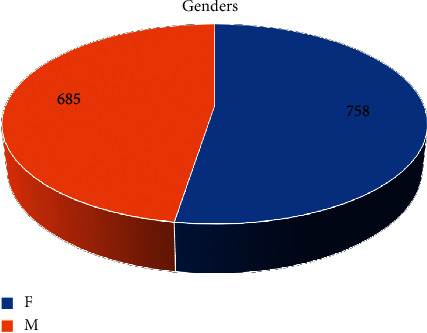
Genders of the patients. The numbers on the pie represent male and female patients in the enrolled population.

A total of 1444 patients were included. The age was distributed from 4–90 years old, which peaked at the range of 50–79 years old (Figure 1). The genders were fairly balanced, with females slightly more than males (758 vs. 685, Figure 2). The majority of the patients were reported as nonHispanic/Latino (Table 1). For races, Caucasians and African-Americans occupied the top two ranks (Table 2).

**Figure 3:**
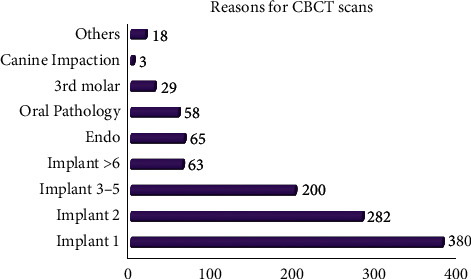
Reasons for CBCT scans. The numbers beside the bars represent the patient counts in each CBCT scan indication.

There were a variety of reasons for taking CBCT scans, and the top four indications were implant treatment planning, endodontic evaluation, oral pathology investigation, and 3rd molar assessment (Figure 3). For implant planning, a single implant contributed to a vast majority of the cases (Figure 3).

**Figure 4:**
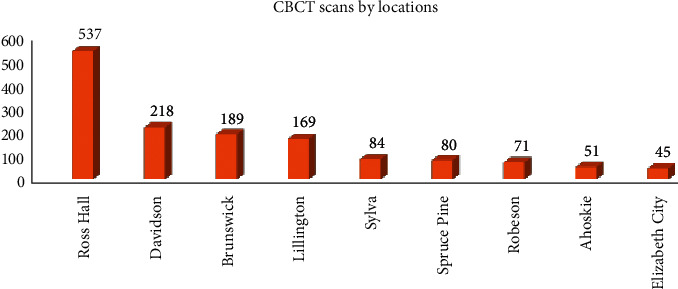
CBCT scans by the locations. The numbers on top of the bars represent the patient counts in each campus location.

For the scan volumes, as expected, Ross Hall acquired the most CBCT scans. Among the eight CSLCs, the top tier, Davidson, Brunswick, and Lillington scanned around 170–220 cases; the middle tier (Sylva, Spruce Pine and Robeson) scanned around 70–85 cases; and the bottom tier (Ahoskie and Elizabeth City) scanned less than 52 cases (Figure 4). A close to 5-fold difference is noted between the top and bottom tiers in terms of scan volume.

Of all the parameters evaluated, only patient age demonstrated normal distribution. One-way analysis of variance and Wilcoxon Rank Sum Test did not reveal significant difference in the demographics, Medicaid coverage, and scan indications among various campus sites (Table 3).

## 4. Discussion

In 2020, there were approximately 57.23 million people, or close to one in five of the total population, living in rural areas of the United States [1]. Rural residents have higher rates of dental caries and tooth loss than the urban population for reasons such as low socioeconomic status, shortage of dental professionals, geographic remoteness, and limited dental insurance coverage [2–5]. A significantly higher number of general dentists and specialists are located in urban relative to rural areas, resulting in a much lower dentist-to-population ration, deficits in dental utilization, and unmet oral healthcare needs in rural areas [6–8].

Many solutions have been proposed to address the oral care demand in rural areas, one of which is to train more dentists that are willing to work in those regions [8], and this brings challenges to US dental schools. A simulation study compared three dental school models—the traditional model, a patient-centered clinic model, and a community-based clinic model—on their ability to attract new dentists and improve dental utilization in rural areas. It was found that the community-based clinics would outperform the other two by retaining more dentists and providing more dental care to the underserved population [9]. This finding corroborates the rationale for recruiting students from rural areas and training students in rural locations to enhance the oral care force in rural areas [8, 10].

In North Carolina, a state with large rural areas, the shortage of oral health caregivers in those regions is aggravating as rural dentists are retiring and leaving the workforce. ECU SoDM was established to alleviate the crisis, with the primary focus of student recruitment being those who desire to stay in rural and underserved areas to provide oral healthcare. The school's CSLCs are unique educational clinics that stretch across North Carolina, offering much-needed dental care to citizens in the surrounding areas, including four counties in the state without a dentist—Camden, Hyde, Jones, and Tyrrell. This education model has been successful, with the school's 357 alumni practicing in North Carolina, many in rural areas.

To ensure the consistent and adequate educational and clinical experience for the students at various campus sites, the patient composition, and dental care demands need to be monitored from time to time. The current study focused on CBCT 3D imaging experience for senior dental students across Ross Hall and eight CSLCs.

It was discovered that the patient demographical profiles, Medicaid coverage, and CBCT indications were comparable across the campus. Most of the scans were referred to patients in an age range of 50–79 years-old, with a peak age of 60–69 (31%), which was similar to what was reported before [11]. The majority of the patients were Caucasian, non-Hispanic/Latino, and without Medicaid.

As far as the reasons for CBCT referrals go, implant planning is the most common indication, followed by endodontics, oral pathology, and 3rd molar evaluation. Among implant cases, one, two, three to five, and more than six implants each accounted for 26.3%, 19.5%, 13.9%, and 4.4% of the overall cases, respectively, with one implant plan being the top reason for CBCT scans. Numerous studies conducted worldwide reveal a similar trend, that the most frequent indication for CBCT scans was for implant site assessment, with the determination of root canal configurations, visualization of impacted teeth, and examination of suspected cysts or tumors being other common reasons for CBCT acquisitions [12–16].

Although the patient composition and CBCT prescription patterns are similar across Ross Hall and eight CSLCs, the quantity of scans taken varied greatly from site to site. Ross Hall had the largest amount of CBCT scans taken, however, they came from various clinics, including D3, D4, AEGD, pediatric graduate clinic, and faculty practice, therefore, the actual cases handled by the D4 clinic may not be that many. Considering CSLC rotation compromises 60% of clinic training in the senior year, the establishment of mixed rotations with high-, intermediate- and low-scan volume sites seem to be critical to warrant consistent and adequate clinical experience for the students on CBCT, implant placement, and other related procedures.

This study was based on a US dental school and dental health care system; however, the implications could have global relevance. The rural–urban disparities and social-economic inequalities in oral healthcare are widespread globally [17–20], urging the need to revolutionize the current dental education system and reallocate available resources to address the unmet oral healthcare demands of underprivileged populations, mostly residing in rural areas. According to the successful model of ECU SoDM, it is speculated that attracting students from rural areas and training them in a rural setting could be an effective strategy to offset the shortage of oral healthcare force in remote communities, not only in the US but in other countries as well.

Although designed as carefully as it could be, there are limitations to the study. The time interval for the investigation was between 2017 and 2021. With population ageing and the alteration of lifestyles, the demand for dental care may be changing. The conclusion drawn from the current study may not be applicable for the years to come. Therefore, a periodic monitoring program would be beneficial to promote the efficacy and effectiveness of the dental curriculum. In addition, the current study focused on the 3D imaging aspect of patient care, and the observation may not be generalized to other dental disciplines, such as endodontic, periodontics, and prosthodontics. Similar across-campus analyses on other dental specialties/procedures are necessary for quality control of dental education and patient care at ECU SoDM.

## 5. Conclusion

The general profiles of the patients and indications for CBCT scans were found to be similar across different campus locations of ECU SoDM. However, the big difference in the amount of scans taken at various sites suggests a well-balanced rotation plan is critical for the successful delivery of dental education and patient care.

## Figures and Tables

**Table 1:** Ethnicity and Medicaid coverage of the patients.

	Yes	No	Nonreported
Hispanic/Latino	71	1130	243
Medicaid	75	1369	N/A

**Table 2:** Races of the patients.

Caucasian	1106
African-American	156
American Indian/Alaskan Native	32
Asian	18
Mixed	13
Other (Native Hawaiian/Pacific Islander, etc.)	73
Nondisclosed	46

**Table 3:** *P* values for the statistical analysis.

Parameters	*P* Values
Age	0.25
Gender	0.41
Ethnicity	0.17
Races	0.23–0.54
Medicaid coverage	0.11
Reasons for the scans	0.21–0.38

“Races” and “Reasons for the scans” had multiple subcategories, and the range of *P* values shown included the whole spectrum of *P* values for all the subcategories. *P* < 0.05 was considered statistically significant difference.

## Data Availability

The data are available upon request.
